# A1 beta-casein milk protein and other environmental pre-disposing factors for type 1 diabetes

**DOI:** 10.1038/nutd.2017.16

**Published:** 2017-05-15

**Authors:** J S J Chia, J L McRae, S Kukuljan, K Woodford, R B Elliott, B Swinburn, K M Dwyer

**Affiliations:** 1 Immunology Research Centre, St Vincent’s Hospital Melbourne, Fitzroy, Victoria, Australia; 2 Freedom Foods Group Ltd, Sydney, New South Wales, Australia; 3 Agricultural Management Group, Lincoln University, Christchurch, New Zealand; 4 Living Cell Technologies, Auckland, New Zealand; 5 School of Population Health, University of Auckland, Auckland, New Zealand; 6 School of Medicine, Faculty of Health, Deakin University, Geelong, Victoria, Australia; 7 Department of Medicine, The University of Melbourne, Melbourne, Australia

## Abstract

Globally type 1 diabetes incidence is increasing. It is widely accepted that the pathophysiology of type 1 diabetes is influenced by environmental factors in people with specific human leukocyte antigen haplotypes. We propose that a complex interplay between dietary triggers, permissive gut factors and potentially other influencing factors underpins disease progression. We present evidence that A1 β-casein cows’ milk protein is a primary causal trigger of type 1 diabetes in individuals with genetic risk factors. Permissive gut factors (for example, aberrant mucosal immunity), intervene by impacting the gut’s environment and the mucosal barrier. Various influencing factors (for example, breastfeeding duration, exposure to other dietary triggers and vitamin D) modify the impact of triggers and permissive gut factors on disease. The power of the dominant trigger and permissive gut factors on disease is influenced by timing, magnitude and/or duration of exposure. Within this framework, removal of a dominant dietary trigger may profoundly affect type 1 diabetes incidence. We present epidemiological, animal-based, *in vitro* and theoretical evidence for A1 β-casein and its β-casomorphin-7 derivative as dominant causal triggers of type 1 diabetes. The effects of ordinary milk containing A1 and A2 β-casein and milk containing only the A2 β-casein warrant comparison in prospective trials.

## Introduction

Type 1 diabetes, one of the most common chronic diseases among children,^1^ is characterised by the selective loss of insulin-producing pancreatic β-cells in genetically susceptible individuals, but a trigger from the environment is generally needed.^2^ The appearance at an early age of autoantibodies directed primarily against one or both of insulin or glutamic acid decarboxylase, but rarely against islet antigen-2, most likely establishes the onset of this disease.^2^ Thereafter, other autoantibodies against either islet antigen-2 or zinc transporter-8 might appear and the more that appear, the greater the risk of rapid progression to clinical disease. However, β-cell autoantibodies may be more representative of reproducible biomarkers of type 1 diabetes pathogenesis and may not be pathogenic themselves.^2^

There are marked variations worldwide in the incidence and prevalence of type 1 diabetes.^3^ Incidence also varies considerably between countries of close geographical proximity that have populations with apparently similar racial/ethnic backgrounds.^3, 4^ The incidence of type 1 diabetes in Iceland is less than half that in Norway, but this difference cannot be explained by known genetic factors because the distributions and frequencies of the known human leukocyte antigen (HLA) class II genes, which affect incidence, are similar in both countries.^5^ Evidence of the involvement of differential exposure to environmental factors comes from studies in monozygotic twins, which suggest that only 13–33% are pair-wise concordant.^6, 7^

An increasing incidence in type 1 diabetes has been observed in most countries. Data from 20 registers in 17 European countries showed a mean increase in children aged <15 years of 3.9% per annum between 1989 and 2003.^8^ The annual rates of increase were generally higher in Eastern European countries (Poland 9.3%, Romania 8.7%, Czech Republic 6.7%) than in western European countries (Spain [Catalonia] 0.6%, Finland 2.4%, Germany [Dusseldorf] 4.7%). However, preliminary evidence from Sweden shows that since 2000, the incidence rate has peaked and started to decline among children aged <15 years.^9^ Recent evidence points to a remarkable increase in China. In Shanghai, the incidence among children aged ⩽15 years increased at a rate of 14.2% per year between 1997 and 2011, from a low baseline of 1.5 per 100 000 in 1997–2001 to 5.5 per 100 000 in 2007–2011.^10^ In Zhejiang, a major city south of Shanghai at an earlier stage of economic development, the mean incidence in adolescents aged ⩽19 years increased at a rate of 12.0% per year, from 1.22 per 100 000 in 2007 (age standardised) to 2.48 per 100 000 in 2013.^11^ The greatest increase in Zhejiang was in children aged <5 years with a rate of 33.61% per year. It is notable that the increasing incidence of type 1 diabetes in China in recent years is mirrored by an increase in per capita dairy product consumption among urban residents of 12 kg from nearly 6 kg in 1992 to 18 kg by 2006.^12^ These findings suggest that environmental factors are significant forces in type 1 diabetes incidence increases.

It is widely acknowledged that genetic and environmental factors interact to precipitate the progression to type 1 diabetes.^2, 13^ Genetic susceptibility factors are well known in terms of the HLA-DR3-DQ2 and HLA-DR4-DQ8 haplotypes, alone or in combination, as reviewed by Pociot and Lernmark.^2^ The contribution of environmental factors is highlighted by: (1) the relatively small proportion of individuals with genetic susceptibility manifesting disease;^14^ and (2) observations that the incidence of type 1 diabetes has increased fastest in recent generations in developed countries.^15^

Various environmental factors have been implicated and include pre- and post-natal exposures,^13^ but we argue that cows’ milk A1 β-casein protein is a key environmental trigger that may explain a significant rise in type 1 diabetes and the different prevalence rates. This does not exclude an effect of other dietary triggers, including gluten/prolamines^16^ and bovine insulin in cows’ milk-based infant formula,^17^ which has been reported to influence insulin autoantibody formation in infants fed a conventional cows’ milk-based formula before 3 months of age.^17^ Changes in modern food processing and storage techniques may also impact:^18^ for example, heat treatment of foods in the presence of sugars (lactose, glucose, fructose) or ascorbic acid, which can produce glycated products, have diabetogenic effects in mouse models.^19^ Furthermore, inhibition of advanced glycation product receptors has been shown to inhibit autoimmune diabetes in mice.^20^

**Figure 1:**
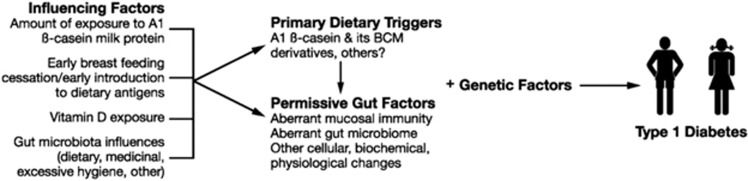
Proposed model for progression to type 1 diabetes in genetically susceptible people.

Permissive gut factors are coexisting mediating mechanisms, which may be involved to varying degrees to favour or enhance the pathogenesis of diabetes (Figure 1) and include factors such as aberrant mucosal immunity, local inflammation and variations in gut permeability.^21, 22^ These may be singularly permissive or interplay as a series of cascading permissive factors that are causally related in sequence.^23^ There are also likely to be many influencing factors involved in responses to dietary triggers, permissive gut factors and progression towards type 1 diabetes, such as short duration/no breastfeeding, caesarean delivery rates and magnitude of exposure to vitamin D (Figure 1).^13, 16, 24, 25, 26^ It is also reasonable to expect that some risk factors common to specific populations in specific geographical locations may be expressed at the population level, while others may only be apparent within populations. Confounder variables, including birth weight, gestational age, Apgar score and maternal age, may also influence disease onset.

Given the pathophysiology of type 1 diabetes (that is, an environmental trigger initiates an autoimmune cascade of events), it is plausible that its incidence can be reduced by removing exposure to individual environmental triggers. This is consistent with the notion that many permissive gut factors and other influencing factors contribute to diabetes outcomes in the presence of a trigger, but are not themselves causal triggers.

### Early exposure to cows’ milk and cereals and other influencing factors for type 1 diabetes

β-cell autoimmunity emerges early in life and early feeding practices may modulate the risk of type 1 diabetes. Indeed, case–control and prospective cohort studies suggest that early childhood exposure to cows’ milk is an influencing environmental risk factor in the development of type 1 diabetes.^27, 28, 29^ This could be a surrogate marker for short or no breastfeeding, and an inverse relationship between breastfeeding and the incidence of type 1 diabetes has been reported.^30^ However, whether the absence of breastfeeding confers increased risk has not been confirmed, because some studies show no effect, others a predisposing effect or a protective effect.^14^ One reason for these diverse results may be that many studies do not distinguish between exclusive or partial breastfeeding (but instead consider ‘overall’ breastfeeding). Furthermore, the duration of exclusive breastfeeding together with the age of introduction of cows’ milk proteins may influence the outcomes. Additional confusion may be introduced by between-country differences in weaning practices, such as weaning to hydrolysed versus intact protein infant formula, or introducing cereal food rather than infant formula as a first infant ‘food’.^31^

If infant age at the introduction to cows’ milk protein is considered together with breastfeeding duration, introducing cows’ milk to infants before 2 months old, compared with 4 months or older is a strong influencing environmental factor for type 1 diabetes (rather than breastfeeding duration per se).^25^ This was demonstrated in a nationwide Finnish case–control study of 690 children with type 1 diabetes (<15 years old), in which univariate analysis showed that type 1 diabetes risk was doubled by the introduction of bovine dairy proteins before 2 months of age.^32^ Furthermore, multivariate analyses of infant age at introduction to dairy and breastfeeding duration showed that early dairy introduction (before 2 months old) was the most important risk factor and that the observed effects of breastfeeding duration in the univariate analysis were explained by their correlation with early bovine dairy introduction in the multivariate analysis.

Early exposure to complex dietary proteins may increase type 1 diabetes-associated β-cell autoimmunity responses in genetically at-risk children, and feeding extensively hydrolysed cows’ milk protein formula (which does not contain intact proteins) may confer better outcomes compared with feeding intact cows’ milk protein formula.^33^ Positivity for two or more autoantibodies is associated with a risk of progression to clinical diabetes of ~60% over 10 years and ~80% over 15 years.^34^ The TRIGR type 1 diabetes primary prevention pilot study of 230 at-risk Finnish children was established to determine whether weaning infants to an extensively hydrolysed-casein milk protein formula versus an 80% intact cows’ milk protein and 20% hydrolysed casein milk protein formula reduced the proportion of children positive for two or more autoantibodies.^25^ Although the pilot study showed that infants weaned to the extensively hydrolysed-protein formula experienced a 50% decrease in the cumulative incidence of β-cell autoimmunity,^25^ this was not sustained in the multinational double-blind randomised clinical follow-up trial of 2159 genetically susceptible infants with an observation period of 7.0 years.^33^ The authors concluded that introduction to an extensively hydrolysed casein versus partially hydrolysed casein cows’ milk protein formula for at least 2 months was insufficient to cause a difference in diabetes-associated autoantibody responses in at-risk infants breastfed for ⩾2 months. However, it is unknown whether an amino acid-based formula or a longer intervention period also has an effect.

Studies investigating an association between cows’ milk consumption and type 1 diabetes have yielded conflicting results. Some studies have not found an association between early exposure to cows’ milk and type 1 diabetes.^31, 35, 36, 37^ This may be explained in part by the interaction between early exposure to cows’ milk-based infant formula and other environmental influencing factors. For example, enteral virus infection is commonly cited as being involved in type 1 diabetes, but it may be the combination of enteral virus infection and early exposure to cows’ milk that is important in determining progression to type 1 diabetes-associated autoimmunity.^38^ Using regression analysis, Lempainen *et al.*^38^ reported a combined effect of enterovirus infection before 12 months of age and early exposure to cows’ milk infant formula (before 3 months) on type 1 diabetes-associated autoantibodies in the Finnish Diabetes Prediction and Prevention Study. Furthermore, differences in cows’ milk proteins, and consequently infant formula protein composition, could influence the findings associated with cows’ milk protein consumption and type 1 diabetes risk.^4^ The magnitude/amount of cows’ milk protein exposure represents another influencing factor as demonstrated in a Finnish case–control study, where children with type 1 diabetes (*n*=33) had a greater likelihood of high milk consumption (>540 ml milk per day) (odds ratio 5.37, 95% confidence interval 1.6–18.4) compared with control children consuming <540 ml milk per day (*n*=254).^29^

Introducing cereal foods before ~3 months of age is also associated with early β-cell autoimmunity,^31^ and the cereal protein gluten has diabetogenic effects in rodents.^39^ Although the practical implications of cereals in infant feeding may be limited because infant feeding guidelines in developed countries do not recommend such early introduction to cereals, Norris *et al.*^31^ suggested that early (<4 months old) and late (⩾7 months old) exposure to cereals was associated with increased risk of β-cell autoimmunity.^31^ This idea of an opportune ‘window’ for certain food introduction has received attention in terms of the best time to introduce allergens to minimise allergy development in at-risk infants.^40^

Recent evidence from Lamb *et al.*^41^ indicates that cows’ milk protein may influence the entire type 1 diabetes disease process. In this prospective Diabetes Autoimmune Study in the Young (DAISY) childhood cows’ milk protein was associated with islet autoimmunity in children with low/moderate genetic risk of type 1 diabetes, but not with high genetic risk. However, once islet autoimmunity was established cows’ milk protein was associated with an increased risk of progression to type 1 diabetes independent of the underlying genetic risk. The timing of introduction of cows’ milk protein was only significant at a very early age for the development of islet autoimmunity. The authors’ conclude that cows’ milk may be diabetogenic when consumed throughout childhood, and may impact both early and later stages of T1D development.

### A1 β-casein milk protein: a primary dietary trigger for diabetes

**Figure 2:**
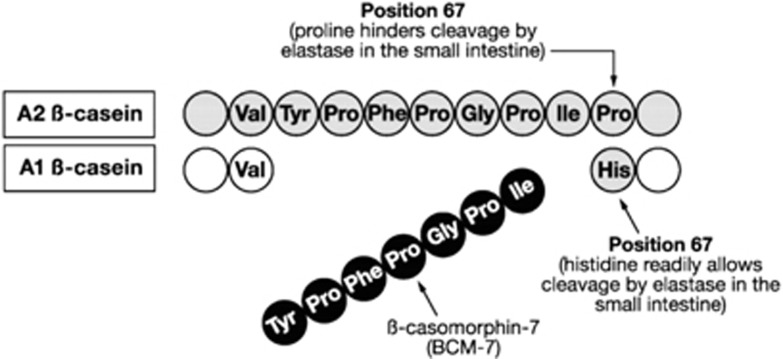
Structures of A1 and A2 β-casein. Adapted from Pal *et al.*^44^

Regional and between-country differences in the incidence of type 1 diabetes are correlated with milk consumption.^42^ One of the main milk proteins is β-casein, which accounts for about 30% of the total protein in cows’ milk.^43^ Several variants of bovine β-casein have been identified, which have differential cleavage patterns *in vivo* stemming from their amino-acid sequences. Cows’ milk contains two major β-casein variants, known as the A1 and A2 types.^44^ These variants differ by a single amino acid at position 67, with a histidine amino acid at this position in the A1 β-casein type and proline in the A2 β-casein type. The histidine residue in A1 β-casein allows cleavage of the preceding seven amino acids, yielding the exogenous peptide β-casomorphin-7 (BCM-7) (Figure 2).^44^ BCM-7 is a μ-opioid receptor agonist^45^ that can cross the gastrointestinal wall and enter systemic circulation.^46^ μ-Opioid receptors are expressed throughout the gastrointestinal tract and elsewhere.^47^ As both the cereal protein gluten and cows’ milk protein A1 β-casein have been implicated as dietary antigens in type 1 diabetes, it is of note that gluten also releases the 7-amino-acid opioid peptide gliadorphin-7.^48^ Gluten has also been associated with increased T-cell reactivity in some patients with newly diagnosed type 1 diabetes.^49^

**Figure 3:**
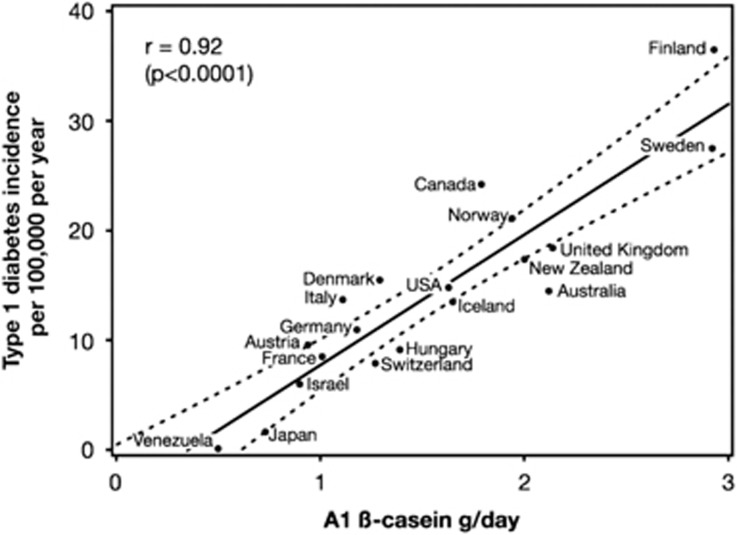
Correlation between A1 β-casein supply per capita in 1990 and type 1 diabetes incidence (1990–1994) in children aged 0–14 years in 19 countries. (*r*=0.92; 95% confidence interval: 0.72–0.97; *P*<0.0001). Dotted lines are the 95% confidence limits of the regression line. Reproduced with permission from R Elliott and The New Zealand Medical Journal (2003).^42^

The proline residue at position 67 in A2 β-casein minimises the likelihood of cleavage. Notably, human breast milk β-casein contains a proline in the homologous position as bovine A2 β-casein protein, so human β-casein is of the A2 type.^50^ Thus, breastfeeding during early infancy eliminates early exposure to A1 β-casein, although BCM-7 derived from dietary bovine A1 β-casein may be transferred to the infant via human breast milk.^51^ Bovine milk A1 β-casein content varies considerably by region, depending on herd genetics. In addition, A1 β-casein consumption correlates significantly with type 1 diabetes incidence and shows a stronger correlation than does milk consumption *per se*.^4, 42^ Laugesen and Elliott examined food consumption data from 19 ‘health care affluent’ developed countries to investigate correlations between food consumption and the rates of type 1 diabetes.^42^ Strong correlations were identified between the consumption of A1 β-casein, but not A2 β-casein, and the incidence of type 1 diabetes. Incidence was highest in Finland and Sweden (countries with the highest A1 β-casein consumption/per capita) and lowest in Venezuela and Japan (countries with the lowest A1 β-casein consumption/per capita) (Figure 3).^42^ The correlation between A1 β-casein and type 1 diabetes was extremely high, with an *r* value of 0.92, the strongest of 15 correlates. Although ecological analyses can be subject to either ecological fallacies and/or confounding, no plausible explanation has emerged in relation to this specific dataset that would invalidate the evidential relationship. With these data, credence is imparted by the very high correlations observed, by the combination of high between-country variation (a factor of 280) with the high variation in the intake of A1 β-casein (more than five-fold variation), and by the high stability of the results to the removal of any individual country from the analyses. Correlations with A2 β-casein (*r*=0.47), latitude (*r*=0.65) and oats (*r*=0.7) were also significant, but only A1 β-casein was significant in multiple regression analyses given the collinearity of these other variables with A1 β-casein. Although it is possible the correlation was created by a third, as yet unidentified common factor, none have been proposed that could explain the high *r* value of 0.92 for A1 β-casein. On the balance of probabilities, A1 β-casein may be a causal factor.

The more recent longitudinal data sets from Shanghai^10^ and Zhejiang,^11^ combined with the threefold increase since 2000 of per capita milk consumption in urban China,^12^ also give credence to a link with a component of milk. Accordingly, the ecological epidemiological data, although not proving causation, provide powerful evidence that A1 β-casein is a causal factor in the pathogenesis of type 1 diabetes.

Although it has been suggested that vitamin D deficiency or the magnitude of exposure to vitamin D are influencing factors that may explain these regional differences in the incidence of type 1 diabetes,^26^ there is limited supporting empirical evidence across populations.^42^ Indeed, Birgisdottir *et al.*^4^ reported similarly strong correlations between lower A1 β-casein consumption and the incidence of type 1 diabetes in Iceland versus four other Scandinavian countries in 2-year-old children, despite all countries sharing similar latitudes, which is relevant to population vitamin D status.

### Animal studies: A1 β-casein as a dietary trigger for autoimmune diabetes

The diabetogenic effects of milk protein were demonstrated in an early study in BioBreeding (BB) rats,^52^ an animal model of spontaneous autoimmune diabetes. Here 50% of rats developed autoimmune diabetes when fed a standard laboratory diet (background rate), which decreased to 15% in rats fed a basic, semi-synthetic diet.^52^ However, when the basic, semi-synthetic diet was supplemented with milk, 52% of rats developed autoimmune diabetes. This rate was 35% in rats fed the gluten-supplemented basic diet.

Since this study, additional animal studies have examined the involvement of A1 β-casein cows’ milk protein and opioid receptors.^53, 54^ In the first of these, non-obese diabetic (NOD) mice were fed a basal diet supplemented with either A1 or A2 β-casein.^54^ While no mice fed the A2 β-casein diet developed autoimmune diabetes, 47% of those fed the A1 β-casein diet developed autoimmune diabetes. Co-administration of the opioid receptor antagonist naloxone attenuated the effects of the A1 β-casein diet, suggesting the diabetogenic effects of the A1 β-casein diet were at least partly mediated via opioid receptors.

The second study was a multicentre trial of BB rats and NOD mice performed by researchers in New Zealand, the United Kingdom and Canada. This study provided limited and inconclusive evidence of differential effects of A1 and A2 β-casein.^53^ The correct interpretation of this study is confounded by implementation events, including infection within the New Zealand mouse colony and feed contamination of both A1 and A2 diets with BCM-7 from partially hydrolysed A1 β-casein. The former was acknowledged within the publication but the evidence of feed contamination with BCM-7 was published only later.^55^

### Human studies

No studies have compared the effects of A1 versus A2 β-casein in milk on the progression towards type 1 diabetes or emergence of associated antibodies in humans. However, β-casein was reported to stimulate T-cell immune responses^56^ and antibody immune responses,^57, 58^ both of which may contribute to the development of type 1 diabetes. Monetini *et al.*^59^ have shown that T-cell lines specific to bovine β-casein can be isolated from the peripheral blood of patients with type 1 diabetes and that these cell lines react with multiple and different sequences of β-casein, particularly towards the C-terminal portion. The same researchers also detected significantly higher levels of antibodies to β-casein in formula-fed infants (*n*=12) under 4 months of age compared with exclusively breastfed infants (*n*=16) (*P*<0.001) and in prepubertal children with type 1 diabetes (*n*=37) compared with age-matched controls (*n*=31) (*P*=0.03).^57^ These findings may represent manifestations of type 1 diabetes and increased sensitivity to antibody reactions. However, in one of the only human studies to investigate the differences in antibody responses to A1 and A2 β-casein, Padberg *et al.*^60^ reported that the ratio of A1 to A2 β-casein antibodies was higher in those with type 1 diabetes than in controls (*P*<0.001). Nevertheless, the significance of these results in genetically at-risk children is open to debate.

Previously, two potential pathways have been suggested as being involved in the link between type 1 diabetes and A1 β-casein: (i) the opioid activity of BCM-7;^54^ and (ii) the similar structures of β-casein and an epitope of the glucose transporter 2 (GLUT-2) expressed on β-cells (that is, immunological cross-reactivity or molecular mimicry).^61^

In the former pathway, opioids like BCM-7 may interfere with metabolic processes, including the regulation of glucose levels and insulin production and these effects are partly prevented by opiate receptor inhibitors such as naloxone.^54, 62^ Such effects may hasten or worsen progression to diabetes. In the second pathway, exposure to A1 β-casein may promote the development of autoantibodies that ultimately contribute to the cascade of events culminating in the development of type 1 diabetes. Autoantibodies to GLUT-2 have been detected in most patients with recent-onset type 1 diabetes^63^ and reactivity of β-casein T-cell lines to human insulinoma extracts and GLUT-2 peptide has been reported.^59^ However, the full implications of these findings are open to speculation because β-cell autoantibodies may not necessarily be pathogenic: rather, they may represent reproducible biomarkers of the pathogenesis.^2^

### Permissive gut factors linking type 1 diabetes and A1 β-casein: intestinal microbiota, gut permeability and mucosal immunity

The current hypothesis for the pathogenesis of type 1 diabetes considers permissive factors interacting at the gut level, including aberrant intestinal microbiota, increased intestinal mucosal barrier permeability and aberrant intestinal immune responses.^22^ During infancy, the presence of commensal intestinal microbiota is critical for various physiologic processes including stimulation of various arms of the innate and adaptive immune systems.^22^ Gut microbiota modulate gut immune function via the innate immune system, such as intestinal epithelial cells and dendritic cells and via the adaptive immune system, particularly intestinal T cells.^64^ The gut immune system may also be involved in type 1 diabetes development via the immunological link between the gut and pancreas.^64^ In fact, NOD mice experience a higher type 1 diabetes incidence in a germ-free environment,^65^ highlighting the environmental impact on gut microbiota outcomes. In humans, low gut microbe diversity of distinct organism types (that is, number, amount and distribution) has been linked to type 1 diabetes and β-cell autoimmunity.^66^

The intestinal surface barrier is an important component of the innate immune system, separating immunogenic material in the intestinal lumen from an immunoreactive submucosa; this may be affected by interactions between aberrant intestinal microbiota and changes in gut barrier permeability.^22^ Previous studies in BB rats using lactulose and mannitol (markers of gut permeability) have shown that the intestine is highly permeable before the onset of type 1 diabetes.^67^ Intestinal myeloperoxidase activities and goblet cell density are also higher in these diabetes-prone rats compared with controls, highlighting a concomitant early intestinal inflammatory response. This is notable because inflammatory mediators can compromise epithelial barrier function and further affect gut permeability. Notably, elevated intestinal myeloperoxidase activity is evident in rodents fed A1 compared with A2 β-casein.^68^ Humans genetically predisposed to type 1 diabetes exhibit intestinal barrier abnormalities.^22^ Because intestinal samples from ‘at-risk’ individuals show gut permeability abnormalities in sugar permeability tests,^69^ these permeability abnormalities may be present before the onset of clinical disease. Similar results have been observed in patients with type 1 diabetes.^70^

Gut permeability abnormalities create opportunities for greater intestinal immune system exposure to dietary antigens such as proteins and peptides, causing altered immune activation and intestinal inflammation.^71^ Although intestinal inflammation is a prerequisite for progression to β-cell autoimmunity and early life impaired barrier function may be an underlying cause of altered responses to gut lumen antigens,^71^ it is unknown whether aberrant responses to antigens cause intestinal inflammation and increased gut permeability or vice versa.

Individually, these factors will be insufficient for promoting the onset of type 1 diabetes, but we propose that one or a combination of these permissive gut factors, together with the A1 β-casein dietary trigger, is generally necessary to precipitate type 1 diabetes in genetically at-risk individuals. The evidence base for the diabetogenic effects of milk, especially A1 β-casein, fits the paradigm of a requisite joint exposure to A1 β-casein plus one or more permissive gut factors (which may also be subject to influencing factors), either concurrently or sequentially, for the induction of type 1 diabetes.

## Conclusions

The evidence for milk and, particularly A1 β-casein, as a primary dietary trigger for type 1 diabetes is intriguing although causation remains unproven. The ecological evidence across populations is particularly strong. Exclusive breastfeeding is widely regarded as being protective against type 1 diabetes in early infancy, but its benefits may be lost if the mother supplements breast milk with cows’ milk formula, or if the duration of breastfeeding is too short. It is also conceivable that some dietary triggers might cross into breast milk. These factors might contribute to the inconsistencies in the reported associations between breastfeeding and type 1 diabetes. Latitude acting as a proxy for vitamin D exposure has been suggested as a potential causal factor, but hypotheses linking vitamin D as a causative trigger in type 1 diabetes have been unrewarding. Thus, we suggest that factors such as vitamin D may act as influencing or modifying factors, but not causal factors. Furthermore, the geographical variability and the chronological changes in type 1 diabetes incidence suggest that changes to influencing factors and permissive gut factors may have altered (for example, gut microbiome profiles), contributing to the increasing incidence of type 1 diabetes.

If A1 β-casein is indeed the dominant causal trigger, then the apparent inconsistent and therefore puzzling results with previous milk studies may be explained. For example, the widespread geographical variation in A1 to A2 β-casein ratio of milk products combined with variable β-casein content of infant formulas, a consequence of different casein to whey ratios of the formulations, may complicate the interpretation of the data.

There are particular challenges associated with prospective studies investigating milk *per se* as causative of type 1 diabetes. These challenges relate to the ubiquity of milk in the diets in developed countries and the long-term nature of any trials. Influencing factors include the potential protective effects of breastfeeding and its duration, and whether or not all bovine milk is excluded from the diet of the mother as well as the baby. However, the potential role of A1 β-casein as a causative trigger for type 1 diabetes could be resolved by prospective studies in genetically at-risk individuals, using milk diets from birth that do not contain A1 β-casein, but which contain β-casein of the A2 type exclusively.
